# Exploring the Origin of the Thermal Sensitivity of Near-Infrared-II Emitting Rare Earth Nanoparticles

**DOI:** 10.1021/acsami.3c04125

**Published:** 2023-06-30

**Authors:** Khouloud Hamraoui, Vivian Andrea Torres-Vera, Irene Zabala Gutierrez, Alejandro Casillas-Rubio, Mohammed Alqudwa Fattouh, Antonio Benayas, Riccardo Marin, Marta Maria Natile, Miguel Manso Silvan, Juan Rubio-Zuazo, Daniel Jaque, Sonia Melle, Oscar G. Calderón, Jorge Rubio-Retama

**Affiliations:** † Department of Chemistry in Pharmaceutical Sciences, Complutense University of Madrid, E-28040 Madrid, Spain; ‡ Department of Optics, Complutense University of Madrid, E-28037 Madrid, Spain; § Nanobiology Group, Instituto Ramón y Cajal de Investigación Sanitaria, IRYCIS, 28034 Madrid, Spain; ∥ Departamento de Física de Materiales, Universidad Autónoma de Madrid, 28049 Madrid, Spain; ⊥ Dipartimento di Scienze Chimiche, Università di Padova, 35131 Padova, Padua, Italy; # Istituto di Chimica della Materia Condensata e Tecnologie per l’Energia (ICMATE), Consiglio Nazionale delle Ricerche (CNR), 35131 Padova, Padua, Italy; ¶ Departamento de Física Aplicada, Universidad Autónoma de Madrid, 28049 Madrid, Spain; ∇ Spanish CRG BM25-SpLine Beamline at the ESRF, 38043 Grenoble, France; ○ Instituto de Ciencias de los Materiales de Madrid-Consejo Superior de Investigaciones Científicas, Cantoblanco, 28049 Madrid, Spain; ▼ Institute for Advanced Research in Chemical Sciences (IAdChem), Universidad Autónoma de Madrid, Madrid 28049, Spain

**Keywords:** rare earth nanoparticles, core@shell@shell, thermometry, photoluminescence emission, NIR, Quantum yield, PL lifetime.

## Abstract

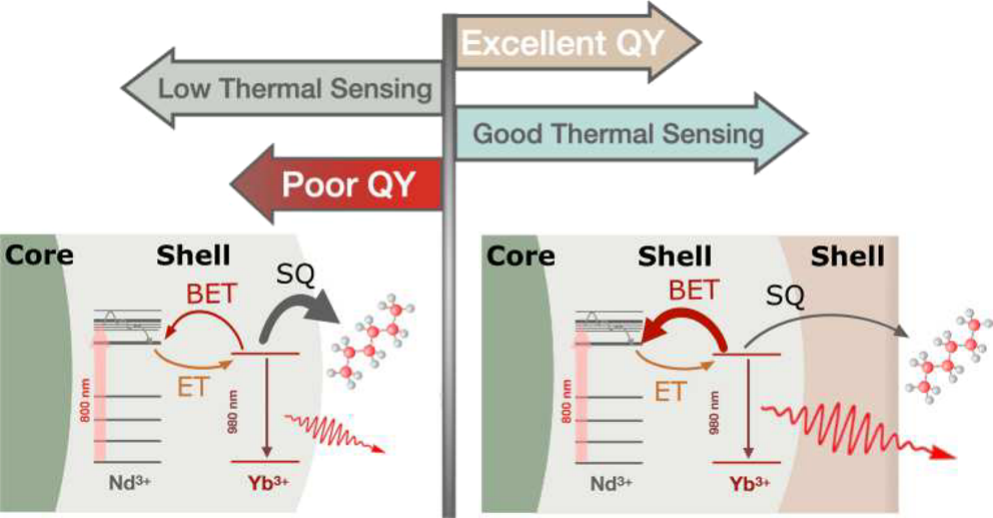

Rare-earth doped nanoparticles (RENPs) are attracting increasing interest in materials science due to their optical, magnetic, and chemical properties. RENPs can emit and absorb radiation in the second biological window (NIR-II, 1000–1400 nm) making them ideal optical probes for photoluminescence (PL) in vivo imaging. Their narrow emission bands and long PL lifetimes enable autofluorescence-free multiplexed imaging. Furthermore, the strong temperature dependence of the PL properties of some of these RENPs makes remote thermal imaging possible. This is the case of neodymium and ytterbium co-doped NPs that have been used as thermal reporters for in vivo diagnosis of, for instance, inflammatory processes. However, the lack of knowledge about how the chemical composition and architecture of these NPs influence their thermal sensitivity impedes further optimization. To shed light on this, we have systematically studied their emission intensity, PL decay time curves, absolute PL quantum yield, and thermal sensitivity as a function of the core chemical composition and size, active-shell, and outer-inert-shell thicknesses. The results revealed the crucial contribution of each of these factors in optimizing the NP thermal sensitivity. An optimal active shell thickness of around 2 nm and an outer inert shell of 3.5 nm maximize the PL lifetime and the thermal response of the NPs due to the competition between the temperature-dependent back energy transfer, the surface quenching effects, and the confinement of active ions in a thin layer. These findings pave the way for a rational design of RENPs with optimal thermal sensitivity.

## 1 Introduction

Rare-earth doped nanoparticles (RENPs) have become one of the most successful systems as photoluminescence (PL) probes due to their photochemical stability, long PL lifetimes, absence of photobleaching, and narrow emission bands.^1−3^ Their multiband emission from the ultraviolet–visible to near-infrared (NIR) spectral range has opened an avenue in lighting applications like sensors,^4,5^ solar cells,^6^ photoresponsive drug delivery systems^7^ or imaging applications.^8−12^ Additionally, some RENPs exhibit temperature-dependent emission: a property that makes them suitable as nanosensors for remote thermal sensing. Luminescent RENPs thermometers have been widely used during the last few years in fields ranging from micro-electronics to in vivo diagnosis and therapy.^13−16^

**Figure 1:**
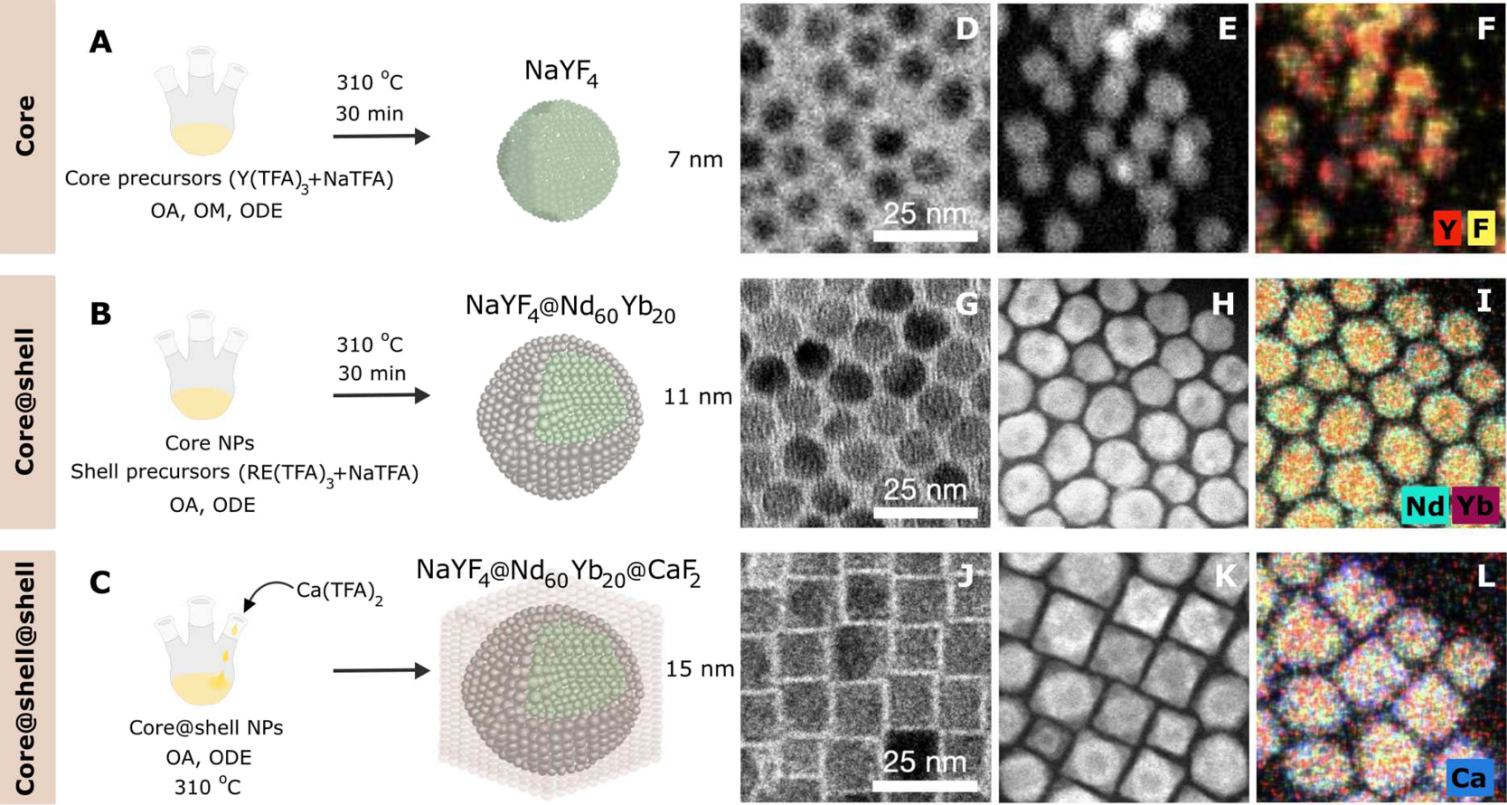
Schematic representation of the inert core (A), core@shell (B) and core@shell@shell RENPs (C) with their representative mean size, composition and synthesis method. (D,G,J) present Transmission Electron Microscopy (TEM) images of core, core@shell and core@shell@shell RENPs, respectively. (E,H,K) and (F,I,L) depict the HAADF-STEM and elemental mapping images of the RENPs obtained at the different steps, respectively.

However, the design of reliable RENPs with high thermal sensitivity and readout precision requires a deep understanding of how composition, structure, and architecture affect their spectroscopic properties and response to temperature changes.^17−19^ One of the most widely known strategies to tune the PL properties of RENPs is the design of core@shell structures.^20^ On one hand, the growth of a protective shell surrounding the RE-ion-doped shell reduces surface defects and vibrational coupling with ligands, thus increasing the PL quantum yield (PLQY) and hence the brightness of the RENPs.^10,11^ On the other hand, confining the ions involved in the PL process in different volumes can increase the versatility and efficiency of the system when compared to core structures. For instance, the presence of a migration energy shell between the absorbing layer and the emitting layer can lead to longer lifetimes;^21^ or isolating two different absorbing ions in separated layers can allow the excitation at two wavelengths.^22^ In summary, an adequate design of both the dopant distribution and the core@shell architecture allowed RENPs to be synthesized with tailored properties. One of the most representative examples is the case of neodymium (sensitizer) and ytterbium (activator) co-doped inert-core@active-shell@inert-shell RENPs (Figure 1) that have been able to yield accurate and absolute thermal images at the pre-clinical level via lifetime thermometry.^18^ Nevertheless, it remains unclear which combination of dopant distributions and core@shell thicknesses leads to structures simultaneously featuring optimal brightness and thermal sensitivity. Such knowledge becomes essential for a proper interpretation of the PL signal and to proceed with further optimization of these structures.

In this work we present a systematic study designed to achieve a complete understanding of the working principles governing the PL and thermal sensing properties of NaYF_4_ inert cores with a neodymium and ytterbium co-doped shell protected with a CaF_2_ external shell. The selection of this external shell is due to its optical transparency in the vis–NIR range, and its minimum lattice mismatch with α-phase RENPs allows its growth on the NaYF_4_ phase. This outer shell heals the surface and minimizes quenching effects due to structural defects and solvent interactions, ensuring in this vein a higher PLQY. Furthermore, its lower electron density when comparing with α-phase NaYF_4_ simplifies its structural characterization under high-angle annular dark-field-scanning transmission electron microscopy (HAADF-STEM), what is of utmost importance. Finally, the possibility of further modification with other molecules like for instance poly(acrylic acid) paves the way to transfer to water for its biological applications. A parametric analysis of structures with different ion distributions and layer thicknesses has been performed to elucidate the advantages of an inert core over an active core, as well as the optimal active shell thickness and the optimal core size on which the shell is grown. Comparison between experimental data and predictions made through a theoretical model highlighted the relevant role of the competition between the thermally activated back energy transfer (BET) from Yb^3+^ to Nd^3+^ ions and the surface-related non-radiative processes. The complete understanding of the physical processes taking place within the RENPs allowed us to synthesize RENPs with high absolute PLQY (≃14%) and elevated relative thermal sensitivity above 1.5% °C^–1^.

## 2 Experimental Section

### 2.1 Chemicals

Ytterbium(III) chloride hexahydrate (99.9%) (YbCl_3_·6H_2_O), yttrium(III) chloride hexahydrate (99.9%) (YCl_3_·6H_2_O), neodymium(III) chloride hexahydrate (99.9%) (NdCl_3_·6H_2_O), calcium chloride (99.9%) (CaCl_2_), sodium trifluoroacetate (NaTFA) (98%), trifluoroacetic acid (TFA) (99%), oleic acid (OA) (90%), 1-octadecene (ODE) (technical grade 90%), oleylamine (OM, >70%), methanol (MeOH) (99.9%), ethanol absolute (EtOH), and *n*-hexane (97%). All the reagents were purchased from Sigma-Aldrich and used as received.

### 2.2 Methods and Characterization

TEM, HAADF-STEM, and energy-dispersive X-ray spectroscopy (EDS) mapping images were taken by a FEI Talos F200X (FEI, USA) operated at 80 kV coupled to an EDS detector. The samples for TEM were prepared by casting a 10 μL drop of each dispersion on a Cu grid with a carbon support membrane. X-ray powder diffraction (XRD) patterns were recorded on a Philips X’pert diffractometer (Cu Kα radiation, 45 kV and 40 mA). Data were collected in the 20–90° 2θ range with a step size of 0.02° and a normalized count time of 1 s step^–1^. The emission spectra were collected under 800 nm CW laser irradiation with an Andor iDus InGaAs 491 cooled to −90 °C. PL decay curves were obtained by exciting the dispersions of RENPs with an OPO oscillator (Lotis) tuned to 800 nm, which provides 8 ns pulses at a repetition rate of 10 Hz. The PL intensity decay curve was detected with a Peltier-cooled photomultiplier tube with enhanced sensitivity in the NIR (Hamamatsu R5509-73). The contribution of scattered laser radiation was removed by using two band-pass filters (FEL850 from Thorlabs) and a monochromator (Shamrock 320 from Andor). The time evolution of the PL signal was finally recorded and averaged by a digital oscilloscope (LeCroy WaveRunner 6000). The absolute PLQY of the RENPs was measured with a 6 in. diameter integrating sphere (Labsphere, 4P-GPS-060-SF). The sample cuvette (5 mm path length) was mounted at the center of the sphere. Light from a pigtailed 808 nm laser (Omicrom, BrixX808-2500-HP-FC) was collimated onto the sample with a beam diameter of 2.5 mm. The collected signal was sent to a monochromator (Horiba, iHR320) for wavelength selection and detected with a NIR InGaAs photodetector (Horiba, DSS-IGA020TC). Hard X-ray photoelectron spectroscopy (HAXPES) measurements were performed at the Spanish CRG beamline BM25-SpLine at the ESRF in Grenoble (France). The set-up consists of an ultra high vacuum chamber, with a base pressure of 1 × 10^–10^ mbar, mounted on a 2S + 3D diffractometer. The chamber is equipped with an electrostatic cylinder-sector analyzer (FOCUS HV CSA) able to handle electron kinetic energies up to 15 keV.^23,24^ Photon energy of 7 and 9 keV, a beam size of 100 × 100 μm^2^, and a fixed incidence angle of 5° were used for the measurements.

### 2.3 Synthesis of α-NaYF_4_@NaYF_4_:Nd_60_,Yb_20_@CaF_2_ RENPs

#### 2.3.1 Synthesis of α-NaYF_4_ Core RENPs

The cores were prepared via thermal decomposition from sodium and yttrium trifluoroacetates (Figure 1A). First, YCl_3_·6H_2_O (1.0 mmol, 303.3 mg) was dissolved in 10 mL of TFA at 90 °C in a three-neck flask. The evaporation of the mixture under a continuous flow of N_2_ yields Y(TFA)_3_ as a white solid powder. This powder was further dissolved in a mixture of OA (3.2 mL), OM (3.2 mL), ODE (6.4 mL), and NaTFA (1 mmol, 136 mg). Subsequently, the solution was heated to 110 °C and kept at this temperature for 30 min, and then to 310 °C and kept at this temperature for 30 min before naturally cooling down to room temperature under an N_2_ atmosphere. The resultant cores were collected through centrifugation (6000 rpm for 5 min, after adding 20 mL ethanol), washed with ethanol (three times), and finally dispersed in 10 mL hexane for further use. The synthesis of active cores doped with Nd^3+^ and Yb^3+^ was carried out as indicated before but using 0.2 mmol of Y(TFA)_3_, 0.2 mmol of Yb(TFA)_3_, and 0.6 mmol of Nd(TFA)_4_, while keeping constant the other parameters.

#### 2.3.2 Synthesis of α-NaYF_4_@NaYF_4_:Nd_60_,Yb_20_ core@shell RENPs

Core@shell RENPs were prepared following a seed-mediated epitaxial growth procedure using the as-prepared α-NaYF_4_ as the cores (Figure 1B). Firstly, rare-earth trifluoroacetate (RE(TFA)_3_) shell precursors were synthesized using the identical procedure for the preparation of the Y(TFA)_3_ precursor above reported, except that 0.2 mmol of YbCl_3_·6H_2_O, 0.6 mmol of NdCl_3_·6H_2_O, and 0.2 mmol of YCl_3_·6H_2_O were used. Second, RE(TFA)_3_ shell precursors were mixed with 10 mL of OA, 10 mL of ODE, 0.5 mmol (68 mg) of NaTFA, and 0.5 mmol (≃90 mg) of core RENPs in a three-neck flask. The mixture was then heated to 120 °C for 30 min, and further to 310 °C for 30 min before a natural cooling down to room temperature. The resultant core@shell RENPs were collected following the same procedure used for α-NaYF_4_ cores and dispersed in 10 mL hexane for further use. Variation of the shell thickness was achieved by modifying the ratios between α-NaYF_4_ cores and RE(TFA)_3_ shell precursors. In this way, we produced 6 samples with shell thickness ranging from 1.1 to 2.8 nm.

#### 2.3.3 Synthesis of α-NaYF_4_@NaYF_4_:Nd_60_,Yb_20_@CaF_2_ core@shell@shell NPs

The procedure for the preparation of the core@shell@shell RENPs is similar to the one for preparing α-NaYF_4_@NaYF_4_:Nd_60_,Yb_20_ core@shell RENPs (Figure 1C). For that, the previously synthesized α-NaYF_4_@NaYF_4_:Nd_60_,Yb_20_ core@shell RENPs were used as cores for a seed-mediated growth of a CaF_2_ shell. In brief, a mixture of α-NaYF_4_@NaYF_4_:Nd_60_,Yb_20_ core@shell RENPs (5 mL, hexane dispersion with a NP concentration of ≃10 mg/mL), 7 mL OA, and 7 mL ODE were first heated to 310 °C and maintained at this temperature under argon gas protection. Subsequently, 1.6 mL of Ca(TFA)_2_ dissolved in OA (0.5 mmol mL^–1^) was injected into the solution divided in eight different injections (0.2 mL each) at intervals of 25 min. The resultant core@shell@shell RENPs were precipitated, washed with ethanol, and finally dispersed in 10 mL hexane.

## 3 Results

Inert-core@active-shell@inert-protective-shell α-NaYF_4_@NaYF_4_:Nd_60_,Yb_20_@CaF_2_ RENPs were synthesized. The α-NaYF_4_ cores have a mean diameter of 7 ± 1 nm as measured by TEM, Figure 1D. HAADF-STEM and elemental mapping images (Figure 1E,F, respectively), show that each core exhibits a homogeneous electron-density and ubiquitous distribution of Y^3+^ and F^–^ within the RENPs. The epitaxial growth of the active shell around the core results in an increment of the RENPs size, as shown in the TEM micrograph, Figure 1G. This active shell is highly doped with Nd^3+^ (60%) and Yb^3+^ (20%) following the composition used in previous works in nanothermometry which has been identified as one with the highest thermal sensitivity.^18^ The different composition between the core and shell is corroborated by HAADF-STEM where an increment of the electron-density can be observed in the outer part of the RENPs, that can be related to an increase of the Z-contrast provided by the Nd^3+^ ions, Figure 1H. Moreover, the elemental mapping reveals the presence of Nd^3+^ preferentially located on the outer part of the RENPs, see Figure 1I. Finally, the incorporation of an inert and protective shell of CaF_2_ is evidenced in Figure 1J where an increment of the size of the RENPs, which correspond to a CaF_2_ shell thickness of ∼2 nm, is observed as well as a modification of the RENPs shape towards a more cubic morphology. Moreover, the HAADF-STEM image shows a variation in the RENPs electron-density (Figure 1K) whereas the elemental mapping reveals the presence of Ca^2+^ ions distributed around the outer shell (Figure 1L). The shape transformation after covering the RENPs with CaF_2_ could be related to a kinetically dominated shell growth regime, due to the lower decomposition temperature of Ca trifluoroacetate compared to the other alkaline-earth trifluoroacetates, which can render a more crystalline structure.^25,26^

To gain insight into the ion composition, EDS and HAXPES analyses of the different RENPs were carried out, see Section S1. All these analyses provide valuable information about the concentration of Nd^3+^ and Yb^3+^ within the matrix and the hierarchical architecture of the core@shell@shell structure. In this vein, HAXPES analysis carried out with a focused X-ray beam at two different photon energies (7 and 9 keV) probes the depth-dependent chemical composition of the RENPs near the surface of the material, where signals arising from the Ca^2+^ ions are predominant (Figure S2). In addition, XRPD analyses performed on the cores, core@shell and core@shell@shell RENPs reveal the crystalline structure, which can be assigned to the α-cubic phase of the NaYF_4_ and CaF_2_, respectively (JCPDS card no. 77-2042), see Figure S3.

**Figure 2:**
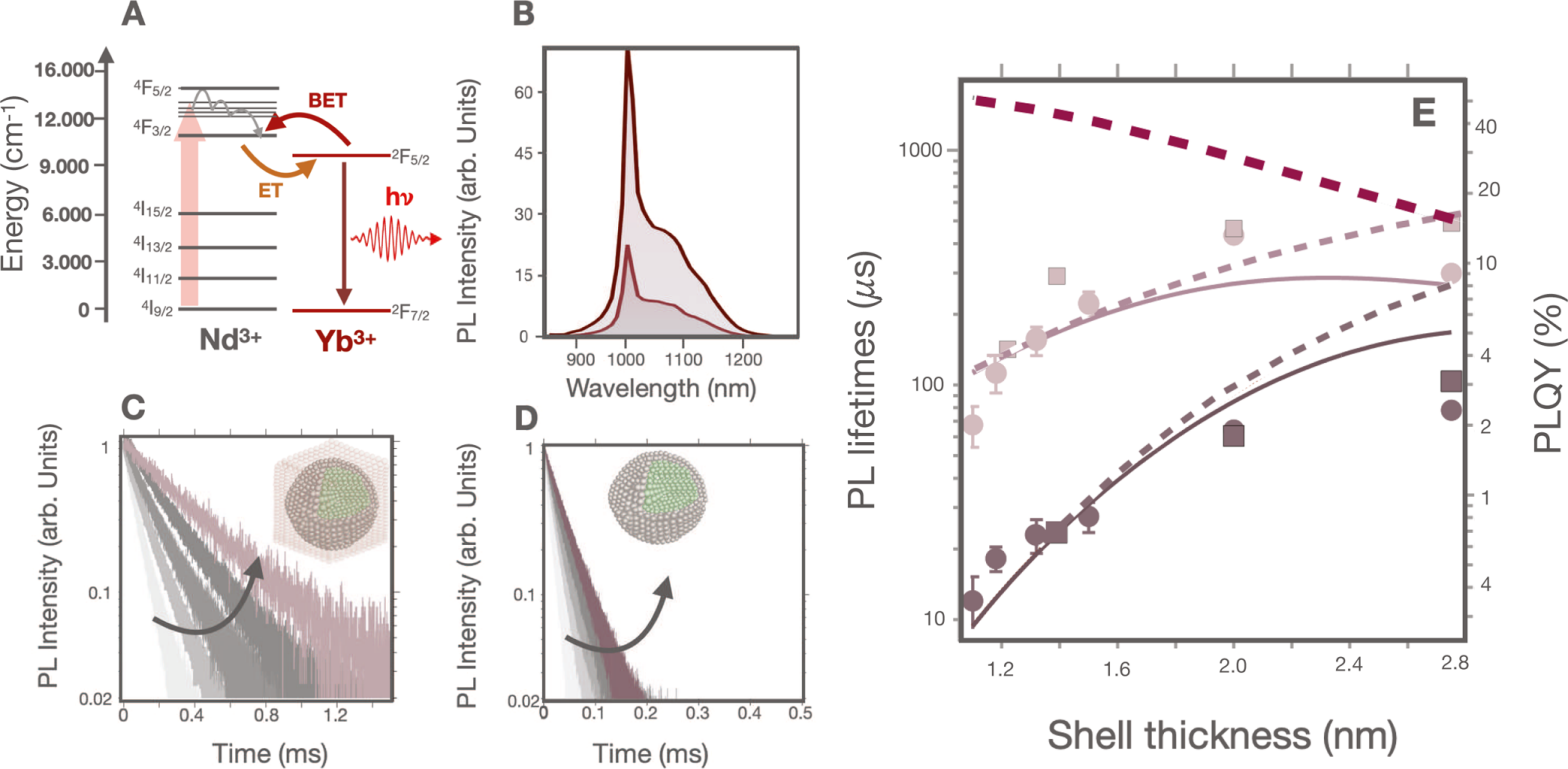
(A) ET scheme for populating the Yb^3+^ 980 nm emission level ^2^F_5/2_ upon excitation of Nd^3+^ ions by a 800 nm laser. (B) Emission spectra of α-NaYF_4_@NaYF_4_:Nd_60_,Yb_20_ (dark gray) and α-NaYF_4_@NaYF_4_:Nd_60_,Yb_20_@CaF_2_ (light gray) RENPs at the same nominal RENPs concentration of 1 × 10^12^ RENPs/mL. (C,D) PL decay curves of α-NaYF_4_@NaYF_4_:Nd_60_,Yb_20_@CaF_2_ and α-NaYF_4_@NaYF_4_:Nd_60_,Yb_20_ RENPs, respectively, when increasing the active shell thickness as indicated by the arrow. (E) Experimental PL lifetime (circle symbols) and absolute PLQY (square symbols) as a function of the active shell thickness for α-NaYF_4_@NaYF_4_:Nd_60_,Yb_20_ (brown), and α-NaYF_4_@NaYF_4_:Nd_60_,Yb_20_@CaF_2_ (pink) RENPs. The dashed gray line represents the theoretical lifetime of the RENPs considering solely host matrix defects as the origin of the emission quenching. The brown and pink dotted lines represent the theoretical lifetimes considering only surface quenching defects, while solid lines represent the theoretical lifetimes when both quenching processes take place.

Figure 2A depicts a schematic energy-level diagram of Nd^3+^ and Yb^3+^ ions within the active shell layer. The figure shows the absorption of the 800 nm excitation beam by Nd^3+^ ions, the ET from Nd^3+^ to Yb^3+^ (and vice versa, BET) and finally the luminescence emission at 980 nm from the excited state ^2^F_5/2_ of Yb^3+^ ions. The energy gap of ∼1000 cm^–1^ between the ^4^F_3/2_ (Nd^3+^) and ^2^F_5/2_ (Yb^3+^) metastable excited states forces a phonon-assisted ET.^27−29^ The thermally activated BET from ^2^F_5/2_ (Yb^3+^) to ^4^F_3/2_ (Nd^3+^) reduces the PL lifetime and the steady state population of the ^2^F_5/2_ of Yb^3+^ causing a decrease of the 980 nm PL emission intensity.^30^ This phenomenon makes the NP emission strongly dependent on the environmental temperature, and it constitutes the working principle of these RENPs as PL lifetime based nanothermometers.

Upon excitation with 800 nm radiation, the core@shell@shell RENPs exhibit a much more intense PL emission than the core@shell RENPs, as shown in Figure 2B, highlighting the importance of the inert outer shell to protect the ions from the large vibrational energies of the solvent and surface-associated ligands.^10,11^

### 3.1 Optimization of the Active Shell Thickness

Based on the structure α-NaYF_4_@NaYF_4_:Nd_60_,Yb_20_@CaF_2_ analyzed in the previous section, we have studied the influence of the active shell thickness on the spectroscopic and thermometric properties of the RENPs, varying the size of this layer from 1.1 to 2.8 nm which is in good agreement with the pioneering study carried out by Tan et al.^18^

The results revealed that PL emission intensity, PLQY, and PL lifetime increased with the active shell thickness. Figure 2C,D show the PL decay curves of α-NaYF_4_@NaYF_4_:Nd_60_,Yb_20_ and α-NaYF_4_@NaYF_4_:Nd_60_,Yb_20_@CaF_2_ RENPs, respectively, for active shell thicknesses from 1.1 to 2.0 nm, in which an increment of the PL lifetime with the active shell thickness is observed. This behavior can be seen in Figure 2E where it can be observed how α-NaYF_4_@NaYF_4_:Nd_60_,Yb_20_ and α-NaYF_4_@NaYF_4_:Nd_60_,Yb_20_@CaF_2_ RENPs show a progressive increment of the lifetime when the active shell thickness is increased until a value of 2.0 nm. Further increase in the shell thickness does not significantly increase the lifetime. Even a slight decrease in lifetime is observed when the RENPs are covered with the protective outer shell. A similar behavior can be observed when we study the PLQY of α-NaYF_4_@NaYF_4_:Nd_60_,Yb_20_ and α-NaYF_4_@NaYF_4_:Nd_60_,Yb_20_@CaF_2_ RENPs as a function of the active shell thickness, Figure 2E. Like in the case of the lifetime, the PLQY increases with the active shell thickness reaching a maximum for shell thicknesses above 2.0 nm. This increase is ascribed to a reduction in the NP surface-to-volume ratio which minimizes energy-loss due to surface quenching (SQ) due to vibrational modes coupled with capping agents or solvent molecules.^11,31^ However, the increment of the active volume increases the number of host defects that can quench the emission^32−34^ and as a result, an optimum shell thickness with a saturation in the thickness-induced improvement is observed when the shell thickness is around 2 nm.

This active layer thickness of 2 nm reduces the energy loss to the surface (which is greater when the active shell is thinner) without significantly increasing the volume of the active matrix and, therefore, without augmenting the non-radiative pathways related to host defects that would increase much more for shell thicknesses greater than 2 nm. Thus, we conclude that the optimum shell thickness is obtained when the sum of both contributions, surface related and host-defect related quenching, is minimized.

To get a deeper understanding of the role played by the different layers of the NP, we developed a simple theoretical model describing the total de-excitation rate of each excited Yb^3+^ ion inside the active layer as a sum of a radiative decay rate Γ_R_ and two non-radiative decay rates attributed to surface effects Γ_S_ and defects in the active region Γ_D_
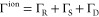
1

Then, the excited Yb^3+^ ion can radiatively decay by emitting a photon with a typical 2 ms radiative decay time τ_R_ = 1/Γ_R_.^35−38^ On the other hand, emission quenching mechanisms can occur through a non-radiative de-excitation of the Yb^3+^ ions, the surface-related quenching processes (Γ_S_) being the most important. SQ effects can be described as a dipole-surface type ET from each Yb^3+^ active ion (donor) to a film of acceptors around the NP. Therefore the ET follows the 1/*d*^4^ distance dependence, the non-radiative ET rate being^35,39−41^
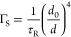
2where *d*_0_ is the Förster distance and *d* is the distance from the Yb^3+^ ion under consideration to the external NP surface. This strong SQ is alleviated by the presence of the external inert shell of CaF_2_. Another non-radiative pathway is the ET process between the Nd^3+^/Yb^3+^ ions and defects present in the active region of the NP (Γ_D_) which is favored by the energy migration between ions. By confining the active ions in a thin layer, the deactivation from the host defects is strongly minimized, as was shown in previous works.^42,43^ Here, this quenching mechanism was phenomenologically simulated by increasing the non-radiative rate Γ_D_ with the volume of the active shell, which accounts for the number of defects in the active region. The maximum value of Γ_D_, which is achieved at the largest thickness used (∼2.8 nm), was taken as three times the radiative value to achieve a plateau for the lifetime in agreement with the experimental results. Then, for the sake of simplicity, we used the following expression
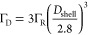
3where *D*_shell_ is the active shell thickness.

The lifetime of each Yb^3+^ ion depends on its position inside the NP following
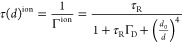
4and also, the quantum yield of the ion
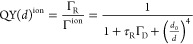
5

To calculate the lifetime of the NP, τ, we averaged the lifetime of all the Yb^3+^ ions, τ^ion^ from eq 4, by weighting each value by the quantum yield of the ion, QY^ion^ (eq 5), which quantifies its emission intensity. To compute the weighted average lifetime, we distributed the Yb^3+^ ions into imaginary thin shells inside the active shell. The ions inside each thin shell are expected to have the same interaction with the surface quenchers.
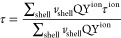
6where *v*_shell_ is the volume of each imaginary thin shell which measures the number of ions inside the shell. In our calculations, the Förster distance *d*_0_ was used as a control parameter to fit the experimental data, its value being 3.6 nm. We calculated the average lifetime τ (see eq 6) for a 7 nm-core NP by varying the active shell thickness from 1.1 to 2.8 nm for RENPs with and without an external inert shell. For the sake of simplicity, we consider a 1 nm thick spherical outer inert shell to roughly represent the variable thickness of the CaF_2_ cubic layer (2 nm in the diagonal directions). The simulated curves are plotted in solid lines in Figure 2E, showing a rough agreement with the experimental data. To analyze the contribution of both non-radiative mechanisms we also plotted in the same figure the lifetime computed when only the SQ is present (brown and pink dotted lines in Figure 2E) and when only the quenching due to defects in the active region participates (dashed gray line in Figure 2E). This clearly shows how the competition between these two non-radiative pathways leads to an optimum active shell thickness close to 2 nm that corresponds to the minimum shell thickness that renders the longest lifetime, and that is in agreement with our experiments.

### 3.2 Inert Core Versus Active Core

As mentioned above, the active ions were confined in a thin layer to minimize the deactivation from the host defects, in accordance with previous works.^18,42,43^ However, we here explore in detail to what extent the confinement of the active ions to a thin layer is advantageous depending on the NP architecture. To answer this question, we synthesized RENPs with active cores (doped with Yb^3+^ and Nd^3+^ ions at the same concentration as in the active shell), following the same synthetic procedure as for the inert core RENPs. As expected, we experimentally found that core@shell@shell RENPs with an inert core show a larger PL lifetime and PLQY than the active core RENPs (Figure S4A,B). However, the situation changes without the protective outer shell: the core@shell RENPs with the inert core presented almost the same optical performance as the active core RENPs (Figure S4A,B). That is, the confinement of active ions in a thin shell is not advantageous without a protective outer shell. This can be ascribed to the strong quenching suffered by the active ions placed in the active shell when no external protective shell is present (see Section S4 for more information).^44^ Once again, this result highlights the importance of covering the NP with a protective CaF_2_ shell. Another relevant issue is the core size of the RENPs that strongly affects the final size of the RENPs which is of utmost importance from an applications point of view. A detailed theoretical study of this point is included in Section S4, showing that RENPs with 7 nm core are optimal, just the ones used in the experiments.

### 3.3 Photoluminescence Emission Temperature Dependence

**Figure 3:**
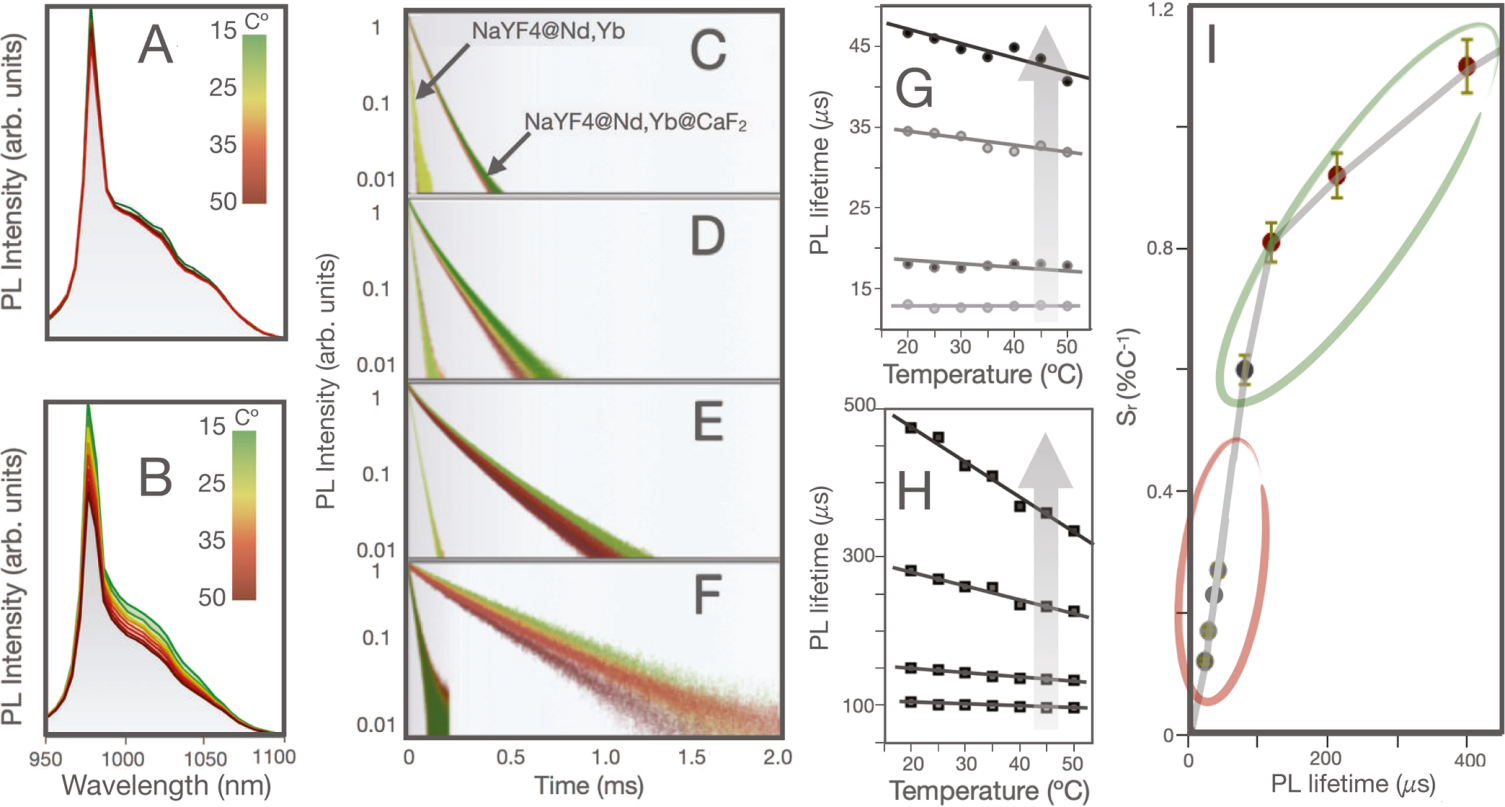
(A) PL emission spectra of α-NaYF_4_@NaYF_4_:Nd_60_,Yb_20_ RENPs and (B) α-NaYF_4_@NaYF_4_:Nd_60_,Yb_20_@CaF_2_ RENPs with an active shell thickness of 2 nm obtained at different temperatures. (C–F) PL decay curves of α-NaYF_4_@NaYF_4_:Nd_60_,Yb_20_ and α-NaYF_4_@NaYF_4_:Nd_60_,Yb_20_@CaF_2_ RENPs with increasing active shell thickness of 1.1, 1.3, 1.5 and 2 nm, respectively. (G) PL lifetimes of α-NaYF_4_@NaYF_4_:Nd_60_,Yb_20_ and (H) α-NaYF_4_@NaYF_4_:Nd_60_,Yb_20_@CaF_2_ RENPs with different active shell thickness as a function of temperature. (I) Relative thermal sensitivity at 15 °C as a function of the lifetime of core@shell (squares) and core@shell@shell (circles) RENPs. The gray line represents a guide for the eye of the trend.

The PL emission of α-NaYF_4_@NaYF_4_:Nd_60_,Yb_20_ RENPs with an active shell of 2 nm varies minimally in the 15–50 °C range, see Figure 3A. In stark contrast, when the same experiment is carried out with α-NaYF_4_@NaYF_4_:Nd_60_,Yb_20_@CaF_2_ RENPs, the PL emission suffers an intense quenching effect while increasing the temperature, as seen in Figure 3B. The same trend can be observed when the PL decay curves of the α-NaYF_4_@NaYF_4_:Nd_60_,Yb_20_ RENPs and α-NaYF_4_@NaYF_4_:Nd_60_,Yb_20_@CaF_2_ RENPs are analyzed, see Figure 3C–F for PL decay curves and Figure 3G,H for the lifetime versus temperature. Thus, when we represent the relative thermal sensitivity  of the different RENPs (see Figure 3I) as a function of the RENPs lifetimes, a clear relationship between both magnitudes is observed. That indicates a definite relationship between thermal sensitivity increase and the RENPs PLQY. For instance, the α-NaYF_4_@NaYF_4_:Nd_60_,Yb_20_ RENPs reach a maximum relative thermal sensitivity *S*_r_ of 0.25% °C^–1^. When the RENPs are additionally covered with an inert shell of CaF_2_ the value of *S*_r_ increases to 1.11% °C^–1^. In fact, the analysis of these data clearly reveals that the thermal sensitivity becomes significant when the RENPs are protected with the outer CaF_2_ shell reaching the maximum thermal sensitivity when the RENPs exhibit the maximum lifetime. Note that the maximum thermal sensitivity was obtained for the RENPs with an active shell thickness of 2 nm. A further increase in the active shell thickness to 2.8 nm reduces the thermal response (*S*_r_ ∼ 0.7% °C^–1^) although the brightness increases (see Section S6).

These results let us hypothesize that the action mechanism that rules the thermal sensitivity of these RENPs is based on a competitive process between emission, the SQ effect and a thermally-dependent BET. Once the RENPs are irradiated with an 800 nm laser, the Nd^3+^ ions absorb the energy and transfer it to Yb^3+^ ions via a phonon assisted process, which populates the excited state ^2^F_5/2_. This state can be depopulated through different ways: (1) by radiative emission of a 980 nm photon, (2) through SQ-which is related to defects, solvents and capping agent vibrational couplings- and (3) through BET from Yb^3+^ to Nd^3+^ ions. According to previous works,^27^ the energy difference between the Yb^3+^ emission barycenter and Nd^3+^ absorption barycenter is Δ*E* ≃ 1300 cm^–1^ and therefore efficient BET would require a lowest number of phonons with a total energy ≃1300 cm^–1^. Following the Dexter model the BET rate can be written as^45^
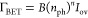
7where *B* is a temperature-independent constant and *n*_ph_ is the temperature-dependent phonon density
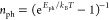
8with *T* the temperature, *k*_B_ the Boltzmann constant, *E*_ph_ the energy of the phonon, and *n* the number of phonons involved in the BET (*n* ≃ Δ*E*/*E*_ph_). As is well known, the matrix NaYF_4_ presents a low phonon energy (∼350 cm^–1^) which makes it especially interesting for RE doped NaYF_4_ upconverting nanomaterials.^46,47^ For NaYF_4_:Er,Yb RENPs the weighted average of the phonon energies was found to be 304 cm^–1^).^48^ Therefore, we took *n* = 4 for our case. *I*_ov_ in eq 7 represents the phonon-assisted spectral overlap between the emission of Yb^3+^ ions and the absorption of Nd^3+^ ions.^49^*I*_ov_ could, in principle, change with temperature due to thermally induced changes in the absorption/emission line shapes, changes in the population of the different Nd^3+^ and Yb^3+^ Stark levels, and thermally induced band shifts. However, previous analyses carried out in the range of temperatures between 200 and 400 K show a reduced variation of the *I*_ov_ value and therefore, in a first order approximation, we could associate the temperature variation of the BET rate to the phonon density *n*_ph_(*T*) (see eq 8). In the previous Sections 3.1 and 3.2, all the experiments were carried out at room temperature and the thermally-activated BET was not taken into account, in accordance with previous works where the BET was activated above 300 K.^49^ Then, in order to study the thermal response of the PL we added to the total de-excitation rate of each excited Yb^3+^ ion Γ^ion^ (eq 1) the decay rate Γ_BET_ (eq 7) by shifting it to zero at the initial temperature (20 °C). Now, if we include the effect of the BET on the lifetime of each Yb^3+^ ion, the expression can be rewritten as
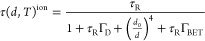
9

The thermal dependence of the Yb^3+^ ion lifetime can be characterized by the relative thermal sensitivity of each Yb^3+^ ion
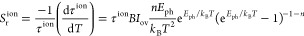
10

Equation 10 demonstrates clearly that a reduction of the temperature-independent non-radiative decay rates (SQ and defects in the active region), which increases the Yb^3+^ ions lifetime, will increase the thermal response, *S*_r_^ion^ ∝ τ^ion^. Therefore, to obtain RENPs with the highest thermal response it is mandatory to increase their PLQY as much as possible.

**Figure 4:**
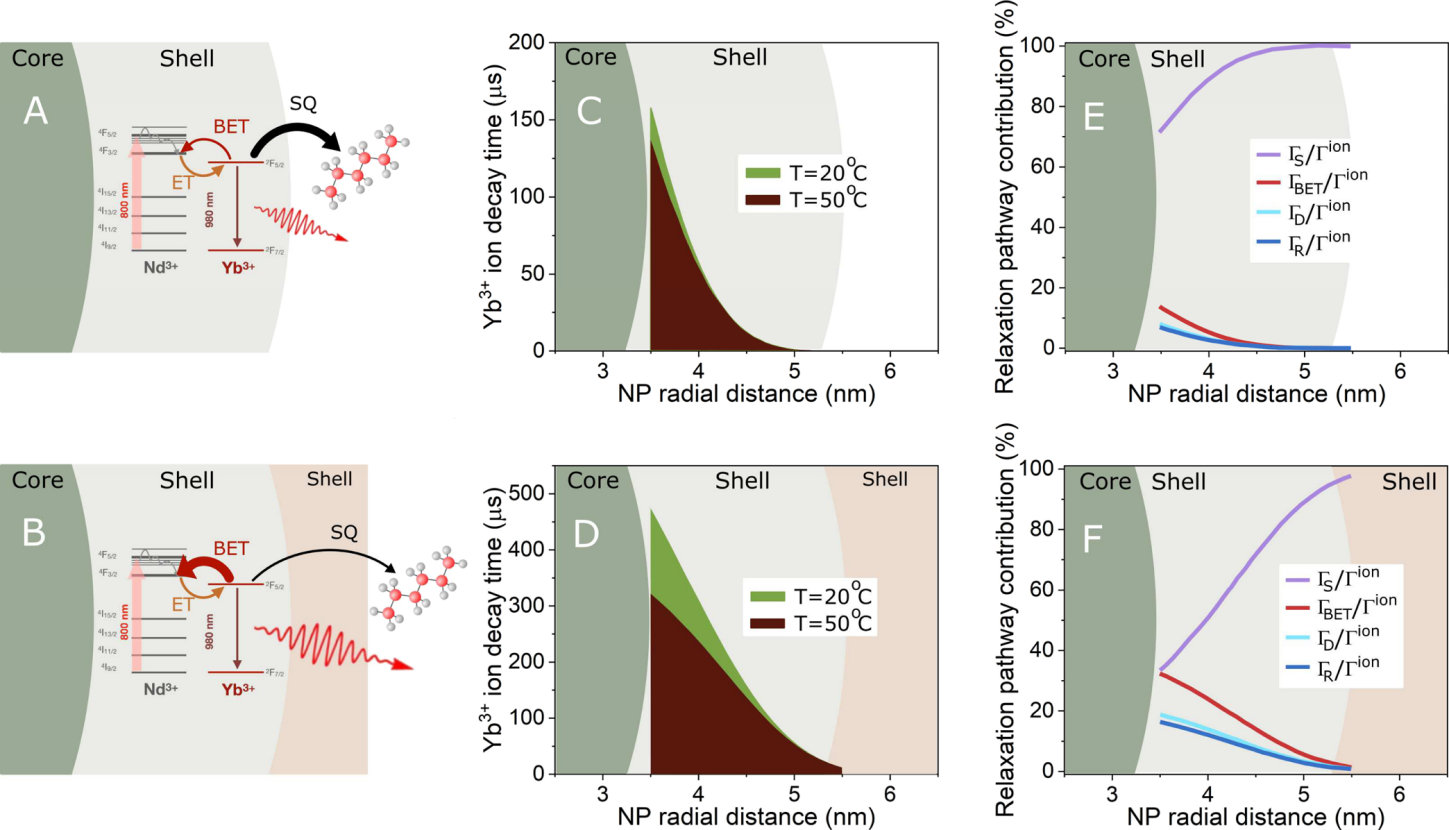
Proposed mechanism of the radiative and non-radiative de-excitation of the Yb^3+^ ions in the absence (A) and in the presence (B) of an inert shell based on CaF_2_. ET stands for energy transfer, BET for back energy transfer, and SQ for surface quenching. Simulation of the radial distribution of the Yb^3+^ ion lifetime for RENPs without (C) and with (D) an outer inert shell at 20 and 50 °C. Simulation of the contribution of the different Yb^3+^ relaxation pathways as a function of the radial position inside the active shell at 50 °C for RENPs without (E) and with (F) an outer shell.

In fact, our experiments have demonstrated that the quenching effect through surface ET seems to be dominant when the CaF_2_ shell is not present. This can be due to its faster non-radiative rate when compared with the radiative rate of Yb^3+^ (∼500 s^–1^). When this non-radiative pathway is dominant, the thermal sensitivity is poor (Figure 4A). In contrast, the presence of the CaF_2_ outer shell significantly reduces the non-radiative pathways due to SQ, increasing the RENPs lifetime and favoring BET from Yb^3+^ to Nd^3+^ which results in an increment of the thermal sensitivity of the RENPs (Figure 4B). Taking into consideration the competition between quenching processes and BET, we have theoretically calculated the lifetime of each Yb^3+^ ion within the structure, τ^ion^, using eq 9 for an active shell of 2 nm. We took a value of *BI*_ov_ = 0.93 s^–1^ to reach a value of Γ_BET_ = 2Γ_R_ at 50 °C. Using these values we obtained a relative thermal sensitivity *S*_r_ close to 1% °C^–1^ in agreement with the experiments (see Figure S7). Figure 4C,D show the τ^ion^ as a function of the radial position inside the active shell for RENPs without (Figure 4C) and with (Figure 4D) the CaF_2_ protective shell at two different temperatures, 20 and 50 °C. Without the outer shell, the lifetime of the Yb^3+^ ions decays steeply with radial distance, due to their proximity to the surface, which facilitates the ET to the SQ defects. As a result, only a few Yb^3+^ ions located in the inner part of the shell are less quenched and can participate in the BET processes overall resulting in RENPs with poor temperature sensitivity (Figure 4C). This scenario changes significantly when the protective inert shell is grown. In this case, the proportion of Yb^3+^ ions quenched is drastically reduced due to the increased distance from the surface quenchers. Consequently, the Yb^3+^ ions located in the inner part of the shell exhibit longer lifetimes and higher thermal sensitivity (Figure 4D). To further indicate how the thermal response is governed by the competition between the BET mechanism and the surface related quenching, we theoretically analyzed the contribution of the different relaxation processes of the Yb^3+^ ions as a function of their position inside the active shell for a temperature of 50 °C. Figure 4E shows the case without the protective shell. The SQ mechanism is clearly dominating for all the Yb^3+^ ions inside the NP, even the ions located in the inner part of the shell where the SQ contributes to 80% of the total decay rate. Figure 4F shows the case with the protective shell. In this case, all the different relaxation processes have a significant contribution, especially for ions placed in the inner part of the shell. This allows the thermally induced BET change to have a large impact on the total decay.

### 3.4 Outer Inert Shell Optimization

**Figure 5:**
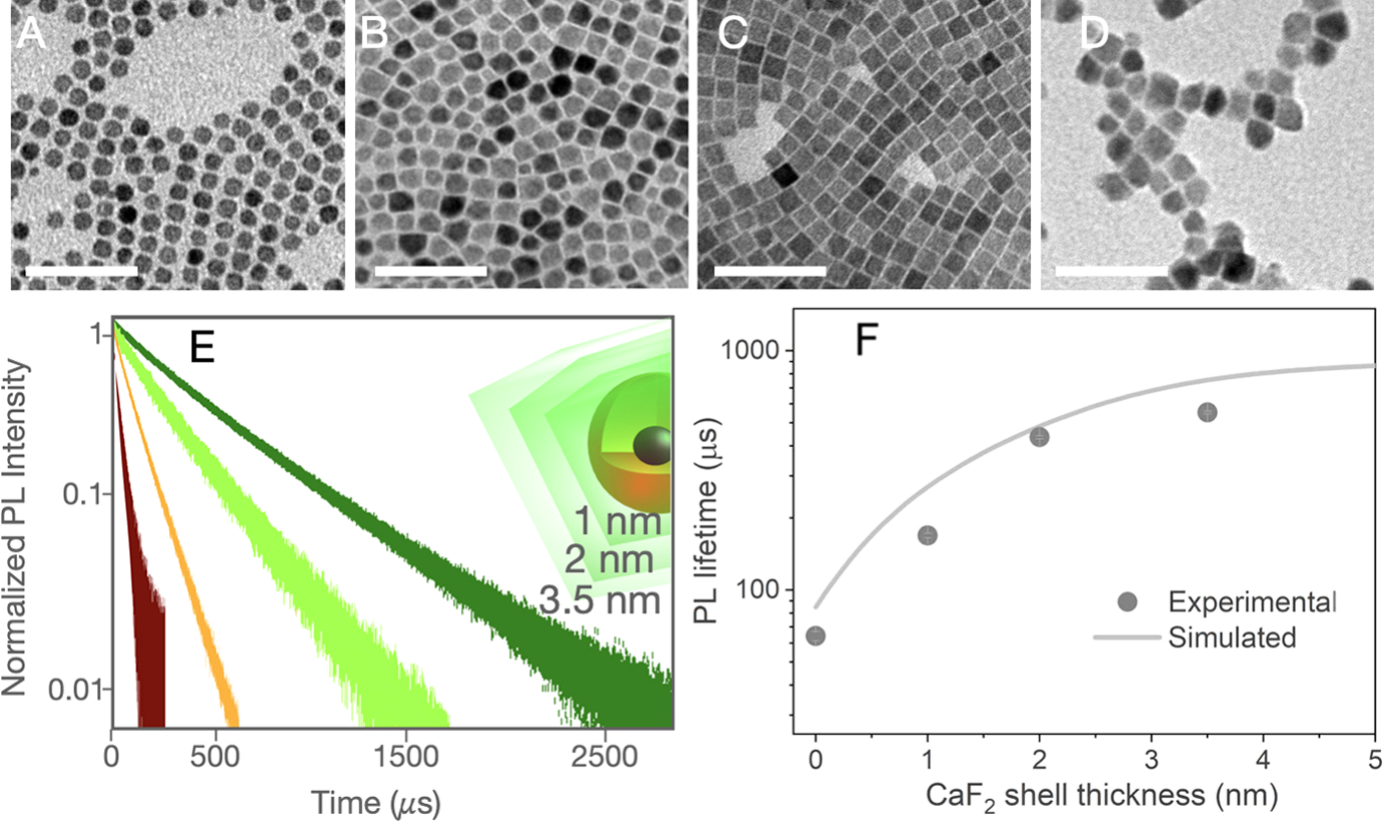
(A–D) present TEM images of the NaYF_4_@NaYF_4_:Nd_60_,Yb_20_@CaF_2_ RENPs with different thickness of the outer CaF_2_ shell: (A) 0, (B) 1, (C) 2, and (D) 3.5 nm. (E) Experimental PL decay curves for NaYF_4_@NaYF_4_:Nd_60_,Yb_20_@CaF_2_ RENPs when increasing the outer shell thickness. Inset shows a schematic representation of the RENPs with different outer shell thickness. (F) Experimental PL lifetime (circles) as a function of the outer shell CaF_2_ thickness. The solid line represents the theoretical lifetime of the RENPs.

The thermal sensitivity of the Yb^3+^ ions is directly related to the ion lifetimes as stated in eq 10 and, therefore, to the ion quantum yields QY^ion^ (eq 5). This observation underscores the importance of reducing the surface-related quenching processes since this reduction would be translated into an enhancement of the thermal sensitivity. Such a reduction can be addressed by growing a thicker inert shell of CaF_2_ which increases the distance from the Yb^3+^ ions to the external NP surface avoiding the SQ phenomena. With this aim, we have synthesized RENPs with a fixed active shell thickness of 2 nm varying the outer inert shell thickness. Figure 5A–D depict the TEM images of the RENPs when the thickness of the outer inert shell is increased from 0 to 3.5 nm. The PL decay curves of these RENPs at room temperature are shown in Figure 5E. As observed, the increment of the CaF_2_ shell thickness produces an enlargement of the PL lifetime (see Figure 5F) that can be directly attributed to a reduction of the SQ processes. This increment of the PL lifetime seems to saturate when the thickness of the outer shell is around 3.5 nm, reaching a value of more than 500 μs. The solid line in Figure 5F represents the theoretical PL lifetime obtained through our model, showing a good agreement with the experimental data. For the RENPs with the thicker outer shell of 3.5 nm, the thermal sensitivity obtained was *S*_r_ ∼ 1.5% °C^–1^ (see Figure S8).

## 4 Conclusions

In this work, we have investigated the PL dynamics of Yb^3+^ and Nd^3+^ co-doped core@shell@shell RENPs to determine the influence of the RENPs architecture on their spectroscopic properties and thermal sensing capabilities. For these reasons, we have synthesized different types of RENPs based on the α-NaYF_4_@NaYF_4_:Nd,Yb structure covered with an outer inert shell of CaF_2_. These RENPs were parametrically characterized to determine the influence of the RENPs architecture on their spectroscopic properties. Thus, we evaluated the influence of the outer inert shell, the thickness of the active middle shell, and the effect of the core size and composition. This information was used to develop a model that describes the radiative and non-radiative processes of the Yb^3+^ ions as a function of their location within the host matrix and of the RENPs architecture. In this model, the temperature-dependent BET is understood as a non-radiative process with a slower rate than other competitive non-radiative processes like surface or host matrix defect at room temperature whereas a comparable rate can be reached by increasing temperature. For this reason, any modification that leads to a reduction of non-radiative processes, like surface or host related quenching, apart from increasing the PLQY, will increase the BET probability and therefore the thermal sensitivity of the RENPs. In this vein, we have experimentally determined that the reduction of the SQ can be accomplished by growing a protective and thick outer shell that avoids ET from Yb^3+^ ions to, e.g., solvent molecules and ligands. On the other hand, the reduction of non-radiative processes related to host defects can be faced by using RENPs where the Nd^3+^ and Yb^3+^ ions are confined in an active thin shell. Furthermore, we have found an optimal active shell thickness of around 2 nm which maximizes the PL lifetime and the thermal response. This architecture has been demonstrated to be a more efficient emitter (PLQY = 14.1%) than RENPs with active cores (PLQY = 8.4%) as long as the NP is covered by an outer shell that alleviates SQ. Finally, we have proved that avoiding surface-quenching effects by increasing the outer inert shell thickness to 3.5 nm produces a further increment in the PL lifetimes that is translated into an augmentation of the thermal sensitivity until 1.5% °C^–1^. This latest result backs our model in which non-radiative processes compete with thermally activated BET, and therefore for obtaining RENPs with higher *S*_r_ it is of outmost importance to get rid of non-radiative relaxation pathways. All of this can be used to envisage the most efficient RENPs suitable for NIR imaging and nanothermometry.

## Supporting Information Available

EDS analysis for core, core@shell, and core@shell@shell NPs, HAXPES analyses for core, core@shell, and core@shell@shell NPs, XRD characterization of the core, core@shell, and core@shell@shell NPs, inert core versus active core, inert core size, active shell thickness and NP brightness, theoretical variation of NP lifetime with temperature, and thermal response for the NPs with a thicker CaF_2_ inert shell (PDF)
