# Supplementary material for: Exploring the Origin of the Thermal Sensitivity of Near-Infrared-II Emitting Rare Earth Nanoparticles
Source: ACS Appl Mater Interfaces. 2023 Jun 30;15(27):32667–77. doi: 10.1021/acsami.3c04125 (PMC10347429; doi:10.1021/acsami.3c04125)
Supplement: Supplementary file 1 — am3c04125_si_001.pdf [file am3c04125_si_001.pdf]

Supporting Information
Exploring the Origin of the Thermal Sensitivity of Near-Infrared-II Emitting Rare Earth Nanoparticles

Khouloud Hamraoui,[†,∇] Vivian Andrea Torres-Vera,[†,∇] Irene Zabala Gutierrez,[†,∇] Alejandro Casillas-Rubio,[‡] Mohammed Alqudwa Fattouh,[†] Antonio Benayas,[¶,§] Riccardo Marin,[¶,§] Marta Maria Natile,[‖,⊥] Miguel Manso Silvan,[#] Juan Rubio-Zuazo,[@,△] Daniel Jaque,[*,¶,§] Sonia Melle,[‡] Oscar G. Calderón,[*,‡] and Jorge Rubio-Retama[*,†]

†*Department of Chemistry in Pharmaceutical Sciences, Complutense University of Madrid, E-28040 Madrid, Spain*

‡*Department of Optics, Complutense University of Madrid, E-28037 Madrid, Spain*

¶*Nanobiology Group, Instituto Ramón y Cajal de Investigación Sanitaria, IRYCIS, Madrid 28034, Spain*

§*Departamento de Física de Materiales, Universidad Autónoma de Madrid, Madrid 28049, Spain*

‖*Dipartimiento di Scienze Chimiche, Università di Padova, 35131 Padova, PD, Italy*

⊥*Istituto di Cimica della Materia Condensata e Tecnologie per l'Energia (ICMATE), Consiglio Nazionale delle Ricerche (CNR), 35131 Padova, PD, Italy*

#*Departamento de Física Aplicada, Universidad Autónoma de Madrid, Madrid 28049, Spain*

@*Spanish CRG BM25-SpLine Beamline at the ESRF, 38043 Grenoble, France*

△*Instituto de Ciencias de los Materiales de Madrid-Consejo Superior de Investigaciones Científicas, Cantoblanco, Madrid 28049, Spain*

∇*Contributed equally to this work*

E-mail: daniel.jaque@uam.es; oscargc@ucm.es; bjrubio@ucm.es

This supporting information includes the following sections:

Section S1. EDS analysis for core, core@shell and core@shell@shell NPs

Section S2. HAXPES analyses for core, core@shell and core@shell@shell NPs

Section S3. XRPD characterization of the core, core@shell and core@shell@shell NPs

Section S4. Inert core versus active core

Section S5. Inert core size

Section S6. Active shell thickness and NP brightness

Section S7. Theoretical variation of NP lifetime with temperature

Section S8. Thermal response for the NPs with thicker CaF_2 inert shell

Section S1. EDS analysis for core, core@shell and core@shell@shell NPs

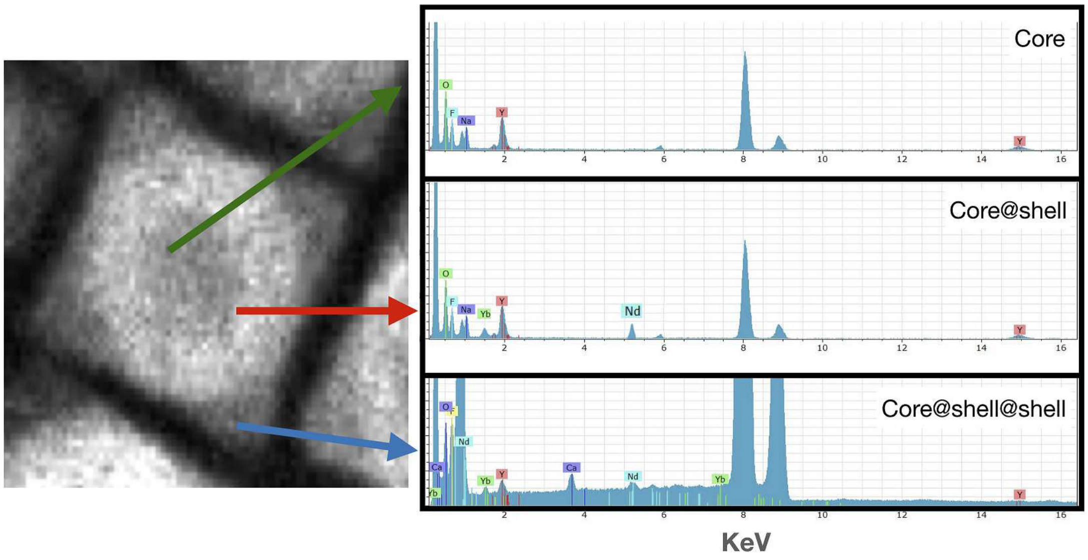

Figure 1: **S1.** EDS analysis.

Section S2. HAXPES analyses for core, core@shell and core@shell@shell NPs

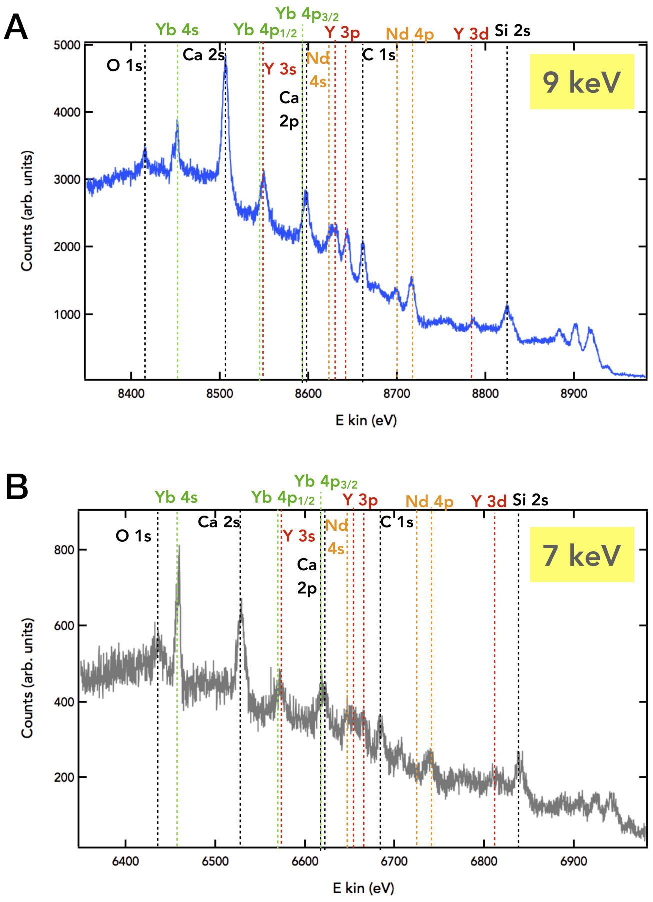

Figure 2: **S2.** HAXPES analyses at two different energies, 9 keV (A) and 7 keV (B).

Section S3. XRPD characterization of the core, core@shell and core@shell@shell NPs

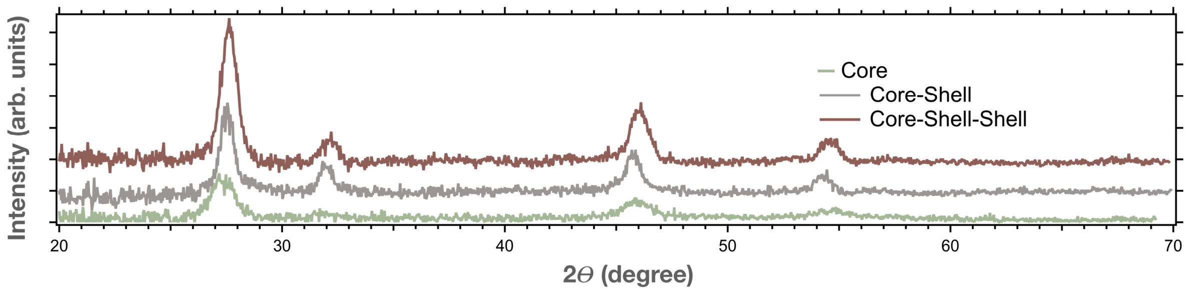

Figure 3: **S3.** XRPD diffraction peaks of the core, core@shell and core@shell@shell NPs. All samples show crystalline peaks corresponding to a $NaYF_4$ and CaF_2 cubic phase structure.

Section S4. Inert core versus active core

We synthesized NPs with active cores following the same synthetic procedure and based on the synthesis of active cores with the same size as the inert cores. After that, an active shell of 2 nm thickness was grown on the cores. We compared the optical performance of these active-core@active-shell NPs with the previously used inert-core@active-shell NPs with a 2 nm-active shell. We found that the active core NPs have the same lifetime than the inert core NPs, as shown in Figure S4A (light colors). The same happens with the quantum yield, as shown in Figure S4B (light colors) where a small increase takes place by doping the cores. Therefore, in the case of core@shell NPs, without the external inert shell, the confinement of the ions in a thin layer does not work which is due to the strong surface quenching suffered by the active ions placed in the active shell. So, although the excitation migration in the case of an active core will produce an overall increase of the non-radiative quenching due to host defects, the Yb^{3+} ions in the core will compensate for the strong surface quenching suffered by the ions placed in the active shell. We corroborated this statement by using our model to compare the decay time τ of a NP with an active shell of 2 nm and an inert core of 7 nm with the same NP with an active core. For the active core NP we took a non-radiative decay rate due to deactivation from host defects equal to $\Gamma_D = 13\,\Gamma_R$ which leads to a value of τ similar to the case with an inert core. Figure S4C shows the radial distribution inside the NP of the decay time of Yb^{3+} ions for both architectures. Here we see how the strong surface quenching suffered by the Yb^{3+} ions placed in the active shell avoids that confined ions show a better performance than the ions placed in the core.

Next, we covered the NPs with the inert shell of CaF_2 which partially alleviates the surface quenching. Now, the inert core NPs present a larger lifetime (see dark colors in Figure S4A) and quantum yield (see dark colors in Figure S4B). In this case the alleviation of the surface quenching produces more efficient Yb^{3+} ions in an active shell with an inert core than the ions distributed in both core and shell which are also quenched by host defects and energy migration. This can be corroborated theoretically in Figure S4D which shows the simulated lifetime of Yb^{3+} ions as a function of the radial distance inside the NP for both structures: inert-core@active-shell@inert-shell and active-core@active-shell@inert-shell. For the active core NPs, we used the same value for the non-radiative rate Γ_D than before without the external shell. Now, the ions confined in the thin shell exhibit large decay times, much larger than the ones corresponding to the ions located in the core for the active core NPs. Then, as expected, the architecture with the active ions doped in a thin shell coated with a protective shell exhibits the best performance.

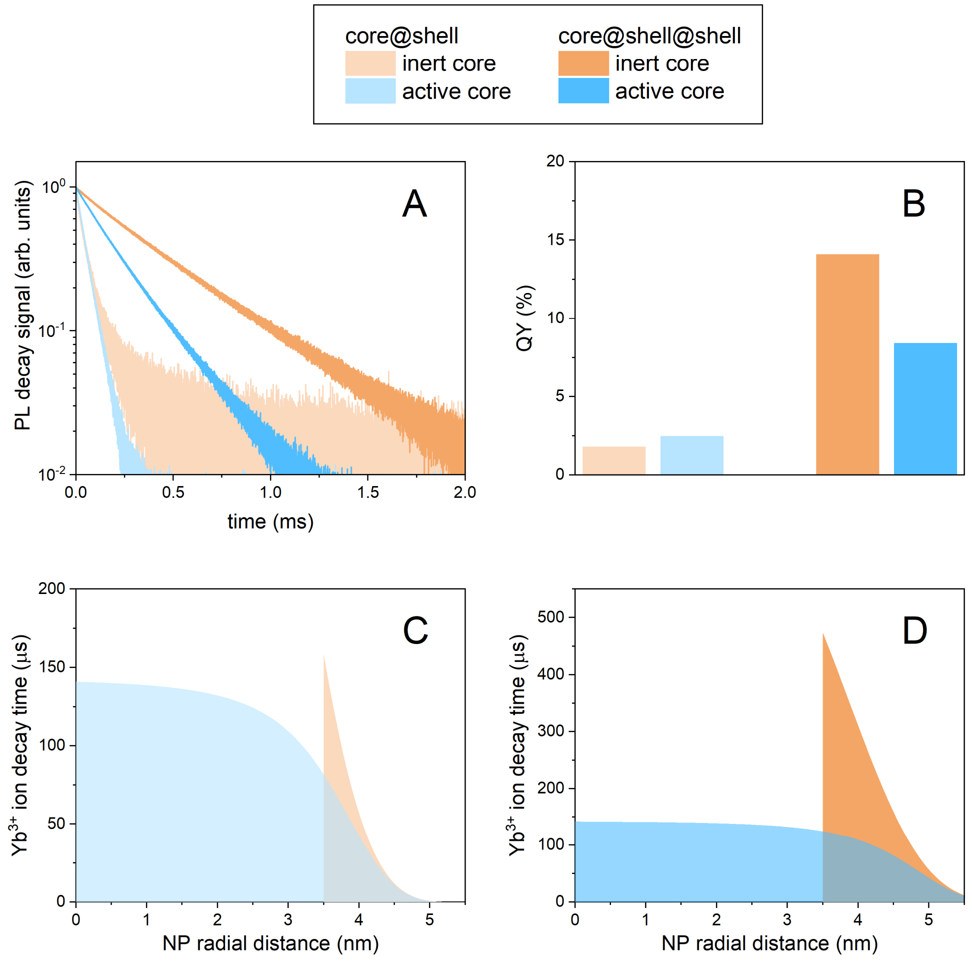

Figure 4: **S4.** A) Experimental PL decay curves and B) PL quantum yield to compare the inert-core NPs (core@shell and core@shell@shell) with the same NPs with active cores (active-core@shell and active-core@shell@shell). Simulated lifetime of each Yb^{3+} ion as a function of the radial distance inside the NP for the case of C) inert-core@shell NPs (light orange) and active-core@shell NPs (light blue) and D) inert-core@shell@shell NPs (dark orange) and active-core@shell@shell NPs (dark blue).

Section S5. Inert core size

One of the most interesting applications of these NPs is their usage as nanothermometers for in vitro and in vivo studies. In this field, the size of the NPs is of utmost importance since it can define the NP pharmacokinetic and therefore its usage.[1] As a general rule, smaller NPs exhibit better biodistribution properties than bigger ones when both are similarly functionalized.[2,3] In this sense, producing the smallest NPs keeping unaltered their spectroscopic properties could be considered as a holy grail. Keeping this goal in mind, we have theoretically evaluated the effect of the core size reduction over the spectroscopic properties of the NPs. However, it should be kept in mind that the reduction of the size core is accompanied with an increment of the surface-to-volume ratio and consequently an increment of the surface deactivation rate.

To discuss this question, we used the previous model and computed the NP lifetime τ by varying the size of the inert core for a NP with an active shell thickness of 2 nm and outer inert shell of 1 nm. The result is plotted in Figure S5A, which shows how the NP lifetime is augmented as the core size increases until a plateau is reached at values close to 7 nm, the value used in our

experiments. This was a surprising result since the change in the inert core size did not affect the non-radiative decay rates present in the active shell, that is, the lifetime of each Yb^{3+} ion should remain unchanged. This can be seen in Figures S5B and S5C (purple lines) where the distribution

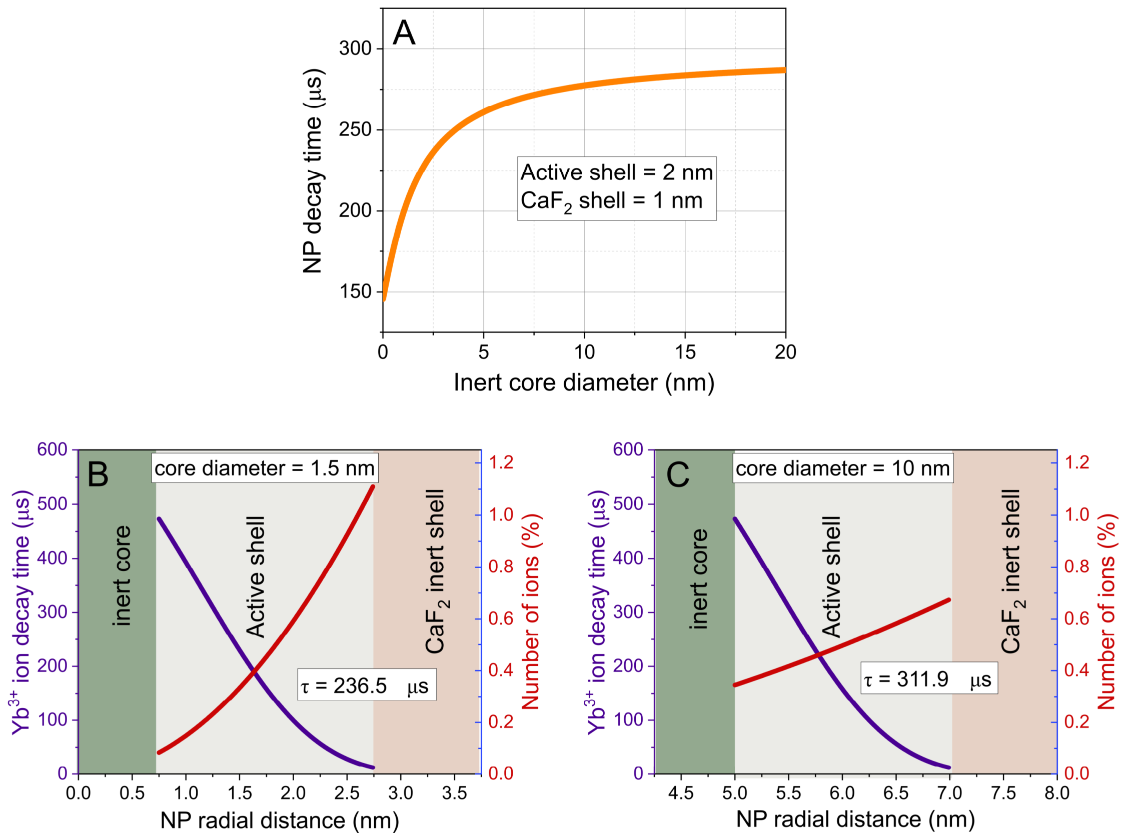

Figure 5: **S5.** A) Simulated representation of the NP lifetime depending on the inert core size. B) and C) Simulated lifetime of each Yb^{3+} ion (left axis) and the number of Yb^{3+} ions (right axis) as a function of the radial distance inside the active shell for the case of an inert core diameter of 1.5 nm (graph B) and 10 nm (graph C). It is clearly shown that the lifetime of each Yb^{3+} ion remains identical for the 1.5-nm core and the 10-nm core NPs (purple lines), while the distribution of the Yb^{3+} ions changes depending on the core size (red lines).

of τ^{ion} along the NP radial distance is plotted for both, a small and a large inert core, respectively. On the other hand, the relative radial distribution of Yb^{3+} ions in the active shell does depend on the size of the inert core. In fact, in a NP with a small core there are many more Yb^{3+} ions in the outer part of the active shell, where the ions are strongly quenched, in comparison to the number of ions in the inner part, where the ions present larger lifetimes (see red line in Figure S5B). By stark contrast, in a NP with a bigger core, a much more uniform distribution of ions with the distance occurs, and then, in comparison, there are more ions with larger lifetimes, as shown in Figure S5C (red line). Therefore, we can conclude that to obtain a more efficient NP the ternary structure core@shell@shell should have a core size larger enough to achieve a nearly uniform distribution of active ions along the radial distance of the NP.

Section S6. Active shell thickness and NP brightness

We analyzed the brightness of core@shell@shell NPs with different active shell thicknesses. In particular we compare the PL spectra of the NPs with 2-nm thickness with the NPs with the maximum thickness used in our experiments, i.e., 2.75 nm. To properly do this comparison, we should normalize the PL spectrum with the concentration or number of NPs in the sample. Therefore, we measured the PL spectrum using the integrating sphere to simultaneously measure the absorbance at the excitation wavelength. The concentration of NPs will be proportional to the absorbance, that quantifies the number of sensitizer ions, divided by the active volume of a NP. Using this magnitude, we normalized the PL spectra and the result is shown in Figure S6. As expected, the NPs with larger active shell thickness have more brightness. However, this is not the case for the relative thermal sensitivity. The maximum value of $S_r = 1.1\%\ ^o\mathrm{C}^{-1}$ was achieved for the NPs with 2-nm thickness whereas the NPs with 2.75-nm thickness present a lower value of $0.7\%\ ^o\mathrm{C}^{-1}$. This reveals that there is an optimal active shell thickness to achieve the maximum thermal response.

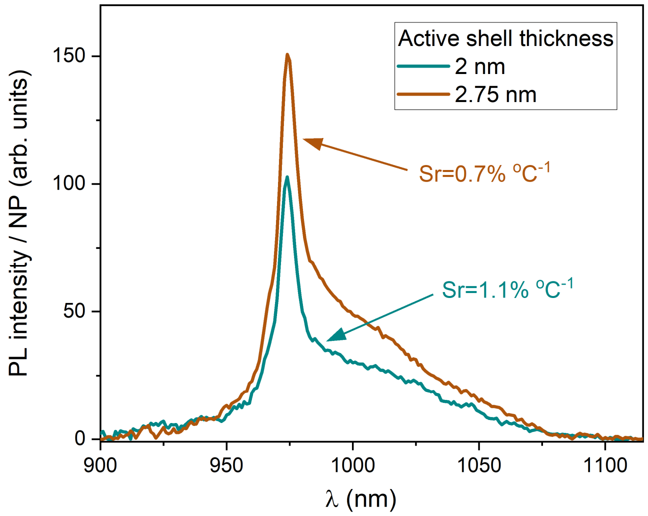

Figure 6: **S6.** PL emission intensity per NP for core@shell@shell NPs with active shell thickness of 2 nm and 2.75 nm.

Section S7. Theoretical variation of NP lifetime with temperature

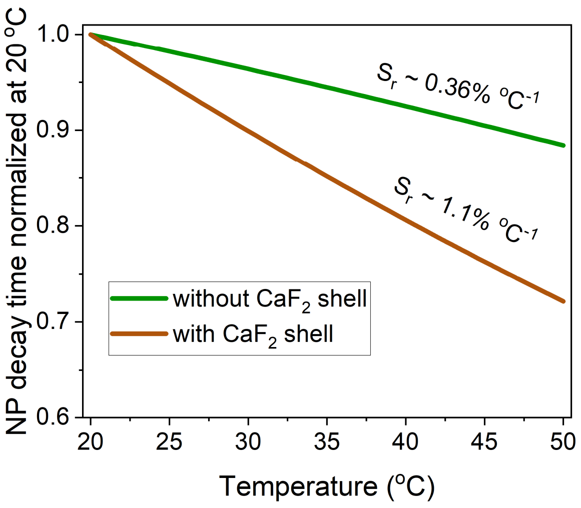

Figure 7: **S7.** Theoretical NP decay time normalized to its initial value at 20 °C as a function of temperature for the case of 2 nm active shell with and without CaF_2 outer shell using the parameter values described in the paper. Using these curves we calculated the relative thermal sensitivity and their values were included in the figure for both structures, showing a roughly agreement with the experimental values.

Section S8. Thermal response for the NPs with thicker CaF_2 inert shell

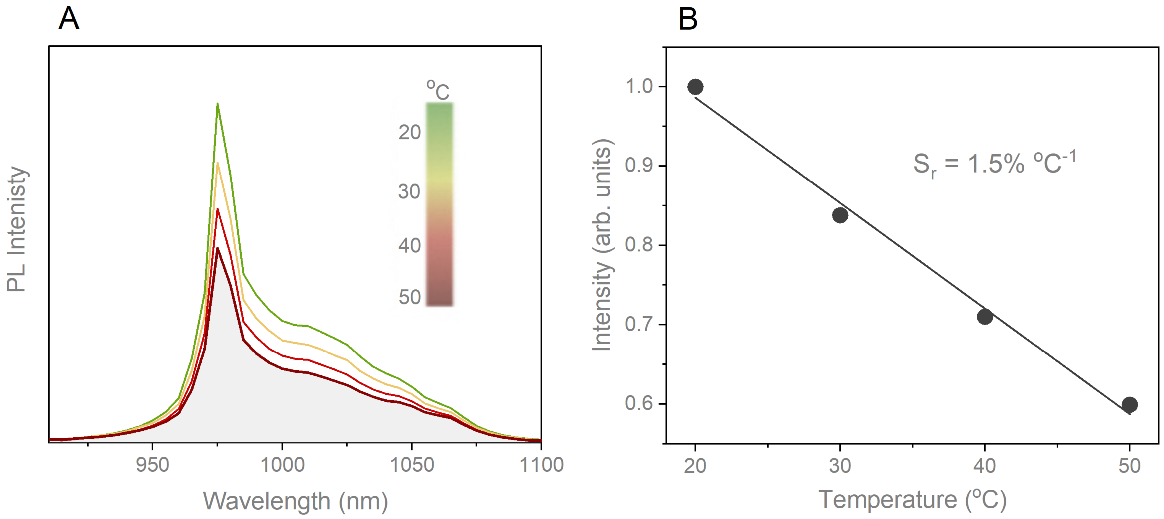

Figure 8: **S8.** PL emission spectra (A) and PL intensity (B) of $NaYF_4$@$NaYF_4$:Nd_{60},Yb_{20}@CaF_2 NPs with an outer inert CaF_2 shell thickness of 3.5 nm as a function of temperature.

References

(1) Hoshyar, N.; Gray, S.; Han, H.; Bao, G. The Effect of Nanoparticle Size on in Vivo Pharmacokinetics and Cellular Interaction. *Nanomedicine* **2016**, *11*, 673–692.

(2) Wang, S. H.; Lee, C. W.; Chiou, A.; Wei, P. K. Size-Dependent Endocytosis of Gold Nanoparticles Studied by Three-Dimensional Mapping of Plasmonic Scattering Images. *J. Nanobiotechnology* **2010**, *8*, 113.

(3) Cruje, C.; Chithrani, B. D. Integration of Peptides for Enhanced Uptake of PEGylayed Gold Nanoparticles. *J. Nanosci. Nanotechnol.* **2015**, *15*, 2125–2131.
